# High Rate of Islets Autoimmunity in Pediatric Patients with Index Admission of Acute Pancreatitis

**DOI:** 10.1155/2023/9170497

**Published:** 2023-11-11

**Authors:** Jonathan D. Tatum, Lindsey Hornung, Melena D. Bellin, Deborah A. Elder, Tyler Thompson, David S. Vitale, Clive H. Wasserfall, Amy S. Shah, Maisam Abu-El-Haija

**Affiliations:** ^1^Division of Pediatric Endocrinology, Cincinnati Children's Hospital Medical Center, Cincinnati, Ohio, USA; ^2^Division of Biostatistics and Epidemiology, Cincinnati Children's Hospital Medical Center Cincinnati, Cincinnati, USA; ^3^Division of Pediatric Endocrinology, University of Minnesota Medical School, Minneapolis, Minnesota, USA; ^4^Department of Surgery, University of Minnesota Medical School, Minneapolis, Minnesota, USA; ^5^Department of Pediatrics, University of Cincinnati College of Medicine, Cincinnati, Ohio, USA; ^6^Division of Pediatric Gastroenterology, Hepatology and Nutrition, Cincinnati Children's Hospital Medical Center, Cincinnati, Ohio, USA; ^7^Department of Pathology, Immunology, and Laboratory Science, University of Florida, Gainesville, USA; ^8^College of Medicine, University of Florida, Gainesville, Florida, USA

## Abstract

**Introduction:**

The underlying pathophysiology of diabetes mellitus after acute pancreatitis is unknown and overall risk of developing diabetes postacute pancreatitis in children is understudied. The objective of our study was to describe the frequency of islet cell autoimmunity and abnormal glucose testing in pediatric patients in the year following their index case of acute pancreatitis.

**Materials and Methods:**

Data were obtained from a single-center observational cohort study of patients with their first episode of acute pancreatitis. Islet cell autoantibody titers were measured on stored plasma collected from acute pancreatitis diagnosis, at 3 months and at 12 months postacute pancreatitis attack. Abnormal glucose testing was defined as the presence of prediabetes or diabetes, as defined by American Diabetes Association criteria.

**Results:**

Eighty-four patients with acute pancreatitis and islet cell autoantibody data were included, 71 had available glucose measures. Median age at first acute pancreatitis attack was 14 years (IQR 8.7–16.3) and 45/84 (54%) were females. Twenty-four patients (29%) were positive for at least one of four islet cell autoantibodies (IAA, GADA, IA-2A, and ZnT8A) and 6 (7%) had two or more positive islet cell autoantibodies. Nineteen patients out of 71 (27%) had abnormal glucose testing at or postacute pancreatitis diagnosis. A higher proportion (37%, 7/19) with abnormal glucose testing had severe acute pancreatitis compared to those with normal glucose testing (13%, 7/52) (*p*=0.04). Patients with normal glucose testing were more likely to be positive for one or more islet cell autoantibodies (31%, 16/52) compared to those with abnormal glucose testing (0%, 0/19) (*p*=0.004).

**Conclusions:**

Islet cell autoimmunity is more common in children after their index acute pancreatitis attack (29%) than in the general population (7%–8%). While the frequency of prediabetes and diabetes postacute pancreatitis is high, other mechanisms besides islet cell autoimmunity are responsible.

## 1. Introduction

Hyperglycemia was historically considered a transient complication of acute pancreatitis with permanent diabetes only occurring after chronic disease as the pancreas undergoes necrosis and fibrosis from long-term disease. Over the last 10 years, diabetes has become an increasingly recognized complication after a single episode of acute pancreatitis in adults [1–4] and there are ongoing efforts to understand the underlying pathophysiology and natural history [5]. Diabetes after acute pancreatitis is likely a heterogenous entity with multiple pathophysiological mechanisms contributing to its development in pancreatitis patients, including insulin resistance, insulin deficiency, altered metabolism of gut hormones and iron, and islet cell autoimmunity [6, 7]. Identifying the underlying mechanisms that lead to diabetes postacute pancreatitis and the type of diabetes is crucial in order to offer the most appropriate therapy for patients. Islet cell autoimmunity has been observed in cases of acute pancreatitis, acute recurrent pancreatitis, and chronic pancreatitis [8–10] but has not been systemically evaluated as a contributing factor in children or adults. Therefore, it is unknown if islet cell autoimmunity through islet autoantibody formation would contribute to diabetes in index acute pancreatitis cases.

While pediatric-specific research has shown an increased prevalence of diabetes in patients with acute recurrent pancreatitis and chronic pancreatitis [11, 12], the frequency of abnormal glucose testing after a single acute pancreatitis attack is understudied. Prior work in pediatric pancreatitis has shown that up to 15% of patients with acute pancreatitis have abnormal glucose testing after their index acute pancreatitis admission with increasing age and body mass index (BMI) being positively associated with abnormal glucose testing [11]. This is similar to adult data, which has shown that the risk of prediabetes and diabetes is approximately 20% for the first 5 years after index acute pancreatitis admission and significantly rises thereafter [2, 3]. Since children have different predominant etiologies of both acute pancreatitis [13, 14] and diabetes [15, 16] compared to adults, it is vital to study the condition in a pediatric-focused cohort. Pediatric studies are needed in order to construct appropriate screening and treatment strategies that will ultimately prevent the long-term morbidity and mortality associated with diabetes.

The goal of our study is to describe the frequency of islet cell autoimmunity and presence of prediabetes and diabetes in pediatric subjects within 1 year of their index acute pancreatitis admission.

## 2. Materials and Methods

**Figure 1:**
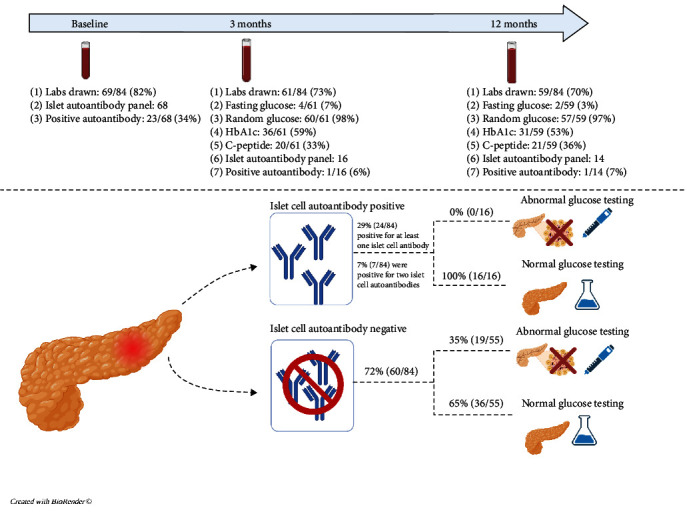
Representation of design of the study, including the number of patients with islet cell autoantibody and glycemic assessments at different time points postindex acute pancreatitis diagnosis. Also displayed is an overview of the results of our study.

Participants in this study were consented and enrolled in an observational cohort registry of patients with index acute pancreatitis admissions (*n* = 89). This study was approved by the institutional review board (IRB 2012-4050), and data are housed in Redcap (Research Electronic Data Capture, Nashville, Tennessee, USA). Two patients who progressed to having acute recurrent pancreatitis at the time of glucose testing, one patient who had a history of a partial pancreatectomy, and two patients who had preexisting type 1 diabetes were excluded from the final analysis. At the time of acute pancreatitis, age, sex, race, ethnicity, BMI, history of prior diabetes, history of insulin use, and coexisting medical conditions were collected. Severe acute pancreatitis, inclusive of moderately severe and severe acute pancreatitis, were defined as per the North American Society for Pediatric Gastroenterology, Hepatology and Nutrition (NASPGHAN) criteria for diagnosis of pediatric acute pancreatitis. Moderately severe acute pancreatitis was defined as “acute pancreatitis with either the development of transient organ failure/dysfunction (lasting <48 hr) or the development of local or systemic complications.” Severe acute pancreatitis was defined as “acute pancreatitis with development of organ dysfunction that persists >48 hr [17].” Laboratory data, including hemoglobin A1C, islet cell autoantibody testing, fasting glucose levels, random glucose levels, and fasting C-peptide levels, were extracted at time of acute pancreatitis diagnosis and captured when repeated at 3 and 12 months after acute pancreatitis diagnosis (Figure 1). All labs were obtained as part of clinical care of patients and per provider discretion and were not part of a research protocol. Baseline labs were collected at the time of admission of first acute pancreatitis diagnosis. The window for the 3-month follow-up visit was between 1 and 6 months postacute acute pancreatitis. The window for the 12-month follow-up visit was between 6 and 18 months after index acute pancreatitis. Prior publications from the acute pancreatitis registry included a subset of study subjects with different goals and objectives than this study [18–21].

### 2.1. Islet Cell Autoantibody Titers

Plasma was collected from blood obtained from subjects within 48 hr of hospital admission with acute pancreatitis, at 3- and 12-month follow-up visits in clinic, then stored under the approved research protocols. Islet cell antibody titers were run on stored plasma kept at −80 Fahrenheit for all participants at the same time. Commercial enzyme-linked immunosorbent assays developed by and purchased from Kronus (Star, Idaho, USA) were used to measure glutamic acid decarboxylase antibodies (GADA), insulinoma-associated-2 autoantibodies (IA-2A), and zinc transporter-8 autoantibodies (ZnT8A) [22]. An in house developed and modified luciferase immunoprecipitation system (LIPS) assay was used to measure insulin autoantibodies (IAA) as described [23]. The laboratory has maintained good accuracy in multiple Islet Autoantibody Standardization Program workshops. In the 2023 workshop with the following thresholds for positivity GADA >5 IU/mL, IA–2A >15 IU/mL, ZnT8A > 20 IU/mL, and IAA >8074 relative light units (RLU) we obtained accuracies of 91%, 88%, 84% and 89%, respectively [24]. These thresholds were used for our study purposes.

### 2.2. Glucose Metabolism Testing

Glucose was measured using a commercially available immunoassay on an Atellica © analyzer (Siemens, Erlangen, Germany). Patients were considered to have abnormal glucose testing if they developed prediabetes or diabetes at any of the follow-up time points after acute pancreatitis diagnosis. American Diabetes Association criteria for prediabetes and diabetes were used (prediabetes: fasting blood glucose between 100 and 125 mg/dL or a hemoglobin A1c between 5.7% and 6.4% (39–46 mmol/mol)); (diabetes: hemoglobin A1c ≥ 6.5% (48 mmol/mol) or fasting blood glucose ≥126 mg/dL) [25]. Oral glucose tolerance testing data were not available for any patients included in the study due to this test not being part of the clinical care of patients post single episode of pancreatitis. C-peptide was measured using chemiluminescent immunoassays performed on the Access 2 Immunoassay system © (Beckman Coulter Life Sciences, Indianapolis, Indiana, USA). A patient was considered to have a low C-peptide if the fasting value was <0.73 ng/mL. The normal range on this assay is 0.73–4.37 ng/mL.

### 2.3. Statistical Analysis

Data were analyzed using SAS®, version 9.4 (SAS Institute, Cary, NC). Due to skewed distributions, continuous data were summarized as medians with interquartile ranges (IQR: 25^th^–75^th^ percentiles), while categorical data were summarized as frequency counts and percentages. Wilcoxon–Mann–Whitney tests were used for group comparisons for continuous data. For categorical data, *χ*^2^ or Fisher's exact tests were used, as appropriate, for group comparisons. A *p*-value <0.05 was considered statistically significant.

### 2.4. Data and Resource Availability

The datasets generated and analyzed in the current study are not publicly available but can be made available upon reasonable request.

## 3. Results

### 3.1. Demographics and Clinical Characteristics

Eighty-four patients with acute pancreatitis median age 14 years, were included in this study and their demographic and clinical data are presented in Table 1.

### 3.2. Islet Cell Autoantibodies

**Figure 2:**
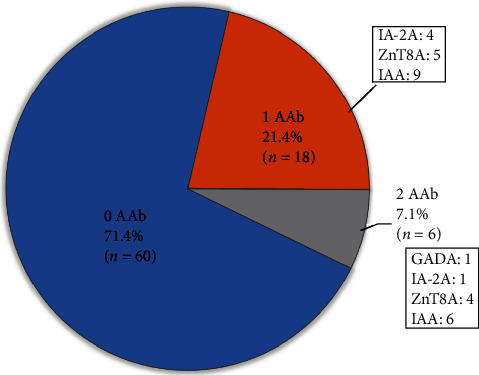
The proportion of patients who were positive for islet cell autoantibodies is displayed. Twenty-one percent were positive for at least one islet cell autoantibody, and 7% were positive for two islet cell autoantibodies. Twenty-three out of 68 (34%) of patients were positive at baseline. Twenty-one of those 23 patients who were positive at baseline did not have islet cell autoantibodies measured at 3 or 12 m. One of the 16 patients was positive for at least one islet cell autoantibody at 3 months and one of the 14 patients was positive for at least one islet cell autoantibody at 12 months.

Twenty-four out of 84 patients (29%) were positive for at least one islet cell autoantibody at any of the three time points (baseline, at 3 months or at 12 months post index acute pancreatitis). Eighteen out of 84 (22%) were positive for a single islet cell autoantibody, and 6/84 (7%) were positive for two islet cell autoantibodies (Figure 2). A vast majority of these patients were positive for at least one islet cell autoantibody at baseline testing (Figure 1). Insulin autoantibody was the most likely antibody to be positive. There were no statistically significant differences between the demographic or clinical characteristics in patients positive for one or more islet cell autoantibodies compared to those who were negative for all four islet cell antibodies (Table 1).

**Figure 3:**
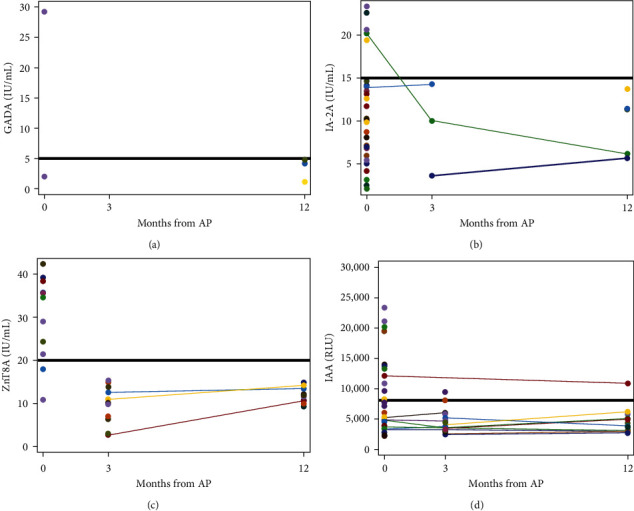
The trend of islet cell antibody titers of the 13 patients with tests at two or more time points over time. Each line represents a single patient and the trend of a specific islet cell autoantibody over the first-year postacute pancreatitis. (a) GADA titers over first-year postacute pancreatitis. (b) IA-2A titers over first-year postacute pancreatitis. (c) ZnT8A titers over first-year postacute pancreatitis. (d) IAA titers over first-year postacute pancreatitis. The dark horizontal line in each panel represents what is considered a positive result (above line) and negative result (below line). Eleven of the thirteen patients (86%) stayed negative, 1/13 (7%) stayed positive, and 1/13 (7%) switched from positive to negative.

Thirteen patients had islet cell autoantibodies tested at two or more time points, allowing an opportunity to evaluate the progression of islet cell autoimmunity over the course of the first year after index acute pancreatitis attack. Eleven of thirteen patients (85%) stayed negative, 1/13 (8%) stayed positive, and 1/13 (8%) switched from positive to negative (Figure 3).

### 3.3. Glucose Metabolism Testing

Seventy-one out of the 84 patients included had glucose testing at any time point and not all patients had glucose testing performed at all time points (Figure 1). Nineteen out of 71 patients (27%) had abnormal glucose testing at any time point within the first-year postacute pancreatitis (Table 2). Overall, there were 19 who were abnormal at either 3 or 12 months (15 and 7 tests were abnormal at 3 months and 12 months, respectively). Of the 19 subjects with abnormal glucose testing, 4/71 (6%) had a diabetes diagnosis and 15/71 (21%) had prediabetes out of the cohort tested. Demographics and clinical characteristics were not different between those who did or did not develop abnormal glucose testing (Table 2). However, a higher proportion (37%) with abnormal glucose testing had severe acute pancreatitis compared to those with normal glucose testing (13%) (*p*=0.04) (Table 2). Patients with normal glucose testing were more likely to be positive for one or more islet cell autoantibodies (31%, 16/52) than those with abnormal glucose testing (0%, 0/19) (*p*=0.004) (Table 2).

### 3.4. C-Peptide Levels

Thirty patients had fasting C-peptide levels drawn at any time point in the first year after index acute pancreatitis admission, five (17%) of whom had low fasting C-peptide values of <0.73 ng/mL. The proportion of patients with low fasting C-peptide levels was not significantly different between those patients who were islet cell autoantibody positive (0%,0/3) and islet cell autoantibody negative patients (19%, 5/27) (*p*=1.00) (Table 1) or those who had abnormal glucose testing (30%, 3/10) and those with normal glucose testing (10%, 2/20) (*p*=0.30) (Table 2). There was no significant difference at any time point between the median C-peptide levels of the patients who were islet cell autoantibody positive (1.5 ng/mL at 3 months; 2.2 ng/mL at 12 months) compared to those who were islet cell autoantibody negative (1.4 ng/mL at 3 months; 1.9 ng/mL at 12 months) (*p*=1.00 at 3 months; *p*=0.59 at 12 months) (Table 1). Finally, there was no significant difference at any time point in the median C-peptide values in those who had normal glucose testing (1.5 ng/mL at 3 months; 1.2 ng/mL at 12 months) compared to those who had abnormal glucose testing (1.2 ng/mL at 3 months; 2.8 ng/mL at 12 months) (*p*=0.22 at 3 months; *p*=0.54 at 12 months) (Table 2).

## 4. Discussion

Our study investigates the frequency of islet cell autoimmunity and abnormal glucose testing in pediatric patients within and after their index case of acute pancreatitis. This is the first study to systemically evaluate the presence of islet cell autoantibodies in patients after they present with their first acute pancreatitis attack. Our study also contributes to the growing body of literature about the prevalence of and clinical characteristics of patients with abnormal glucose testing after a single episode of acute pancreatitis.

The frequency of islet cell autoantibody positivity in our pediatric cohort (29%) was much higher than the general healthy population, which has been reported to be between 7% and 8% [25, 26]. We observed that multiple autoantibodies were present in 7% of our patients. Compared to the general healthy population without diabetes, in which 0.8% have two or more islet cell autoantibodies positive [25], our prevalence was much higher (about ten times the general population). This finding is of significance given that patients with multiple autoantibodies present have a 50% chance of developing diabetes at 6 years of seroconversion and a >80% chance at 12 years [27]. While autoimmunity was not associated with abnormal glucose testing within our short follow-up period (12 months) postacute pancreatitis, acute pancreatitis may trigger islet cell autoimmunity that portends an increased risk of developing diabetes later in life. We observed transient islet cell autoantibody positivity that resolved over the first year after acute pancreatitis diagnosis in a single patient, which suggests that acute inflammation may elicit an immune response to islet cell antigens that wanes over time as the pancreatic inflammation subsides, at least in a subset of patients. A similar phenomenon has been observed in a single adult male patient who developed GAD autoantibody positivity that resolved within 1 year after acute pancreatitis diagnosis [9]. Transient elevations of single islet cell autoantibodies have been well-described in relatives of patients with type 1 diabetes and are considered to have low predictive value of future development of type 1 diabetes, although no definitive conclusions have been made about their clinical relevance [28–30]. The presence of islet cell autoimmunity has been observed with recurrent episodes of acute pancreatitis in both children and adults and our frequency is similar to what has previously been reported in recurrent pancreatitis [8, 12, 31]. Yadav et al. [10] in a recent pilot study of one hundred adults with acute recurrent pancreatitis and chronic pancreatitis reported 35% of patients were positive for at least one islet cell autoantibody and 7% were positive for multiple. Belin et al. [12] reported islet cell autoantibody positivity in 38% of the thirteen pediatric patients with acute recurrent pancreatitis and chronic pancreatitis tested. While these studies suggest recurrent inflammation of the pancreas may trigger islet cell-directed autoimmunity in a subset of patients, our findings suggest that only one episode of acute pancreatitis may be sufficient as a trigger for islet autoimmunity.

Despite the high frequency of islet cell autoimmunity, we did not find an association with islet cell autoimmunity and the development of abnormal glucose testing over the first-year postacute pancreatitis. In fact, we found that patients positive for at least one islet cell autoantibody were statistically more likely to have normal glucose testing than abnormal glucose testing. This lack of association between islet cell autoantibody positivity and abnormal glucose testing suggests other factors outside of an autoimmune process are the key drivers in dysglycemia that occurs within the first-year postacute pancreatitis attack.

The existing literature suggests that multiple mechanisms are likely contributing factors, including insulin resistance mediated by proinflammatory cytokines, insulin deficiency, altered metabolism of gut hormones, and iron [6, 7]. We found that severity of acute pancreatitis mediated a greater likelihood of developing abnormal glucose testing. The course of severe acute pancreatitis could elicit a greater degree of inflammation, leading to cytokine-mediated insulin resistance. Insulin deficiency alone seems an unlikely cause in our cohort. Our study did not find a significant association with low C-peptide levels and abnormal glucose testing and did not find a difference in the median C-peptide values between those with normal and abnormal glucose testing. However, the sample size was small. These C-peptide findings contrast with most adult studies, which have found elevated C-peptide levels in patents with diabetes after index acute pancreatitis case [32, 33]. The relatively high BMI of our cohort, with 39% of patients meeting criteria for overweight or obesity, which is similar to the most recently reported prevalence of overweight and obesity [34], may suggest weight-related insulin resistance is a factor in the development of abnormal glucose testing. However, there are likely other factors besides BMI contributing to abnormal glucose testing (e.g., genetics) in some patients after their first episode of acute pancreatitis since there was no statistically significant difference between the BMI of patients with normal glucose testing as compared to abnormal glucose testing. Overall, our results highlight the lack of definitive knowledge about and the complexity of postacute pancreatitis diabetes and suggest that likely diabetes in pancreatitis subjects has multifactorial etiologies. Prospective studies dedicated to elucidating the underlying pathophysiology of diabetes postacute pancreatitis are clearly needed.

The frequency of prediabetes or diabetes after a single episode of acute pancreatitis that we observed is higher than previously reported in pediatric studies [11] and similar to adult studies examining glucose homeostasis over the first-year postacute pancreatitis attack [1–3]. As previously stated, severity of pancreatitis appeared to mediate an increased risk of abnormal glucose testing within the first-year postacute pancreatitis in our patients. A similar finding has been reported in pediatrics [11], while adult literature is inconsistent regarding this relationship [3, 35]. Age, BMI, and race/ethnicity were not associated with abnormal glucose testing, which is similar to adult findings [3]. In the future, defining the subtype or subtypes of diabetes that can occur after a single episode of acute pancreatitis is critical, so treatment plans based on the underlying pathophysiology can be implemented.

Despite this being the largest cohort of pediatric patients to have their islet cell autoantibodies and glucose homeostasis studied after a single acute pancreatitis attack, our study has limitations. Our sample size is relatively small, and the study sample comes from a single center. Therefore, our results may not yet be generalizable. Our study design limits us from determining if islet cell autoimmunity was present before the initial testing performed at the time of acute pancreatitis diagnosis, but this is the case in real-life acute pancreatitis scenarios. Additionally, there were a high number of patients who did not have glucose-related data collected at each time point of our study, which may have contributed to inconsistency in our results. These incomplete data occurred because of patients missing scheduled appointments and lab draws as is typical in clinical care, the context in which the labs were initially obtained. Finally, our short follow-up period of 1 year precludes us from identifying patients whose abnormal diabetes testing resolved or, conversely, those patients who may develop diabetes years later. Given that we have a prospective cohort design, longer follow-up may be needed to define temporal changes.

## 5. Conclusion

In conclusion, we found a high rate of islet cell autoantibody positivity in patients within a year of index acute pancreatitis admission, with these patents being ten times more likely to be positive for one or more islet cell autoantibody compared to the general population. We also found an increased rate of prediabetes and diabetes postindex acute pancreatitis compared to the existing pediatric and adult literature. Islet cell autoantibody positivity in our cohort was not associated with abnormal diabetes testing within 12 months post-acute pancreatitis, which suggests other factors besides islet cell autoimmunity may play a role in the development of diabetes after first acute pancreatitis attack. Future larger, prospective studies are needed to further our understanding of the pathophysiology and time course of diabetes after index acute pancreatitis attack in order to provide knowledge to inform screening guidelines and optimal treatment.

## Figures and Tables

**Table 1:** Demographics and clinical characteristics of patients with islet autoantibody positive status.

	All islet testing *n* = 84	Positive for any islet autoantibody titers *n* = 24 (29%)	All four islet autoantibodies negative *n* = 60 (71%)	*p*-value ^*∗*^
Age at 1^st^ AP attack (years)	14.0 (8.7–16.3) (1.5–20.4)	14.9 (8.8–17.0) (3.8–19.5)	12.7 (8.7–16.0) (1.5–20.4)	0.36
Sex (female)	45 (54%)	14 (58%)	31 (52%)	0.58
Race White/Caucasian Black/African American Other	75 (89%) 7 (8%) 2 (2%)	19 (79%) 4 (17%) 1 (4%)	56 (93%) 3 (5%) 1 (2%)	0.13
Ethnicity (non-Hispanic/Latino)	81 (96%)	24 (100%)	57 (95%)	0.55
BMI percentile	69.7 (22.5–96.6) *n* = 79 (0.0–99.8)	56.6 (15.8–91.8) *n* = 21 (0.0–99.3)	73.0 (24.0–97.1) *n* = 58 (0.0–99.8)	0.37
BMI ≥ 85^th^ percentile	31/79 (39%)	6/21 (29%)	25/58 (43%)	0.24
SAP during AP episode	16 (19%)	3 (13%)	13 (22%)	0.54
Comorbid conditions	49/83 (59%)	13 (54%)	36/59 (61%)	0.57
Exocrine insufficiency at 1^st^ attack	0/80 (0%)	0/23 (0%)	0/57 (0%)	1.00
Insulin use prior to AP	0 (0%)	0 (0%)	0 (0%)	1.00
Pre-DM/DM any point up to 12 months post AP	19/71 (27%)	0/16 (0%)	19/55 (35%)	**0.04**
Low C-peptide up to 12 months post AP (<0.73 ng/mL)	5/30 (17%)	0/3 (0%)	5/27 (19%)	1.00
Time AP to abnormal C-peptide (years) up to 12 months post AP	1.0 (0.6–1.0) *n* = 5	–	1.0 (0.6–1.0) *n* = 5	–
C-peptide 3 months (ng/mL)	1.4 (1.1–2.0) *n* = 20	1.5 (1.3–1.7) *n* = 2	1.4 (1.0–2.1) *n* = 18	1.00
C-peptide 12 months (ng/mL)	1.9 (0.9–2.9) *n* = 21	2.2 (1.2–3.3) *n* = 2	1.9 (0.7–2.9) *n* = 19	0.59

Data presented as median (25^th^–75^th^ percentile) (min–max) or *n* (%).  ^*∗*^*p*-values for testing positive versus negative groups. Variables with missing data are noted with “*n* = ” or “/*n*” (denominator indicating how many had data) if not noted then full data were available. AP, acute pancreatitis; BMI, body mass index; SAP, severe acute pancreatitis; DM, diabetes mellitus. Bold values denote the statistical significance *p*-value.

**Table 2:** Clinical characteristics and glucose metabolism data of patients with normal and abnormal glucose testing.

	Normal testing for DM post AP *n* = 52 (73%)	Abnormal testing (pre-DM or DM) post AP *n* = 19 (27%)	*p*-value
Age at 1^st^ AP attack (years)	14.1 (8.8–16.7) (1.5–19.5)	14.6 (8.7–17.1) (4.0–20.4)	0.75
Sex (female)	28 (54%)	11 (58%)	0.76
BMI percentile	71.4 (22.5–96.4) *n* = 50 (0.0–99.8)	72.9 (22.2–97.2) (3.1–98.9)	0.99
BMI ≥ 85^th^ percentile	21/50 (42%)	8 (42%)	0.99
SAP during AP episode	7 (13%)	7 (37%)	**0.04**
Any positive islet cell autoantibodies at baseline or 3 months or 12 months	16 (31%)	0 (0%)	**0.004**
Multiple positive islet cell autoantibodies at baseline or 3 months or 12 months	4 (8%)	0 (0%)	0.57
Low C-peptide (<0.73 ng/mL)	2/20 (10%)	3/10 (30%)	0.30
Time AP to abnormal C-peptide (years)	1.1 (1.0–1.2) *n* = 2	0.6 (0.2–1.0) *n* = 3	0.40
C-peptide 3 months (ng/mL)	1.5 (1.3–2.0) *n* = 12	1.2 (0.7–2.0) *n* = 8	0.22
C-peptide 12 months (ng/mL)	1.2 (0.9–2.2) *n* = 13	2.8 (0.7–3.5) *n* = 8	0.54

Data presented as median (25^th^–75^th^ percentile) (min–max) or *n* (%). Variables with missing data are noted with “*n* = ” or “/*n*” (denominator indicating how many had data) if not noted then full data were available. AP, acute pancreatitis; BMI, body mass index; SAP, severe acute pancreatitis; DM, diabetes mellitus. Bold values denote the statistical significance *p*-value.

## Data Availability

The datasets generated and analyzed in the current study are not publicly available but can be made available upon reasonable request.
